# Dose-Response Effects of Acute Aerobic Exercise Intensity on Inhibitory Control in Children With Attention Deficit/Hyperactivity Disorder

**DOI:** 10.3389/fnhum.2021.617596

**Published:** 2021-06-18

**Authors:** Yu-Jung Tsai, Shu-Shih Hsieh, Chung-Ju Huang, Tsung-Min Hung

**Affiliations:** ^1^Department of Physical Education and Sport Sciences, National Taiwan Normal University, Taipei, Taiwan; ^2^Department of Psychology, Northeastern University, Boston, MA, United States; ^3^Graduate Institute of Sport Pedagogy, University of Taipei, Taipei, Taiwan; ^4^Institute for Research Excellence in Learning Science, National Taiwan Normal University, Taipei, Taiwan

**Keywords:** intensity, acute exercise, executive function, arousal level, ADHD

## Abstract

The present study aimed to examine whether the effect of acute aerobic exercise on inhibitory control of children with attention-deficit/hyperactivity disorder (ADHD) is moderated by exercise intensity. Using a within-subjects design, 25 children with ADHD completed a flanker task with concurrent collection of electroencephalography (EEG) data after three different intensities of treadmill running. The results showed that low- and moderate-intensity exercises resulted in shorter reaction times (RTs) relative to vigorous-intensity exercise during the incompatible condition of the flanker task regardless of task congruency. A P3 congruency effect was observed following low- and vigorous-intensity exercises but not after moderate-intensity exercise. The mean alpha power, a measure of cortical arousal, increased following low- and moderate-intensity exercises but decreased following vigorous-intensity exercise. In addition, the change in arousal level after moderate-intensity exercise was negatively correlated with RT during incompatible flanker tasks. The current findings suggest that children with ADHD have better inhibitory control following both low- and moderate-intensity exercises relative to vigorous aerobic exercise, which could be characterized by an optimal state of cortical arousal.

## Introduction

A deficit in executive function is one of the core cognitive deficits in attention-deficit/hyperactivity disorder (ADHD) (Willcutt et al., 2005), a condition which can persist from early childhood to young adulthood (Biederman et al., 2011; Lin and Gau, 2018). In particular, deficits in inhibitory control in children with ADHD have been considered as an endophenotype (McAuley et al., 2014). Inhibitory control refers to withholding the attention of an individual in response to task-irrelevant but prepotent information and stays focused on goal-relevant information (Barkley, 1997). Children with ADHD have demonstrated weaker inhibitory control signified by longer reaction times (RTs) and lower levels of accuracy during neuropsychological testing, compared with typically developing children (Mullane et al., 2009). Impaired inhibitory control has been associated with failures in several cognitive processes such as attention allocation, stimuli evaluation, as well as action monitoring (Doehnert et al., 2010; Liotti et al., 2010; Kratz et al., 2011). Given that inhibitory control is one of the cornerstones of complex cognitive operations (e.g., cognitive flexibility and planning) (Miyake et al., 2000), and that deficits in this cognitive domain are one of the neuro-pathologies in children with ADHD (McAuley et al., 2014), understanding approaches that facilitate inhibitory control may be of significance.

A growing body of evidence has demonstrated a positive effect of acute aerobic exercise on inhibitory control in children with ADHD (Chang et al., 2012; Pontifex et al., 2013; Chuang et al., 2015; Piepmeier et al., 2015). In particular, previous studies suggested that moderate-intensity aerobic exercise, defined either as 50–70% of heart rate reserve (HRR) (Chang et al., 2012; Chuang et al., 2015; Yu et al., 2020) or 60–75% of maximal HRR (Pontifex et al., 2013; Piepmeier et al., 2015), facilitates inhibitory control in children with ADHD. However, the effect of low (<40% HRR) and vigorous-intensity (>70% HRR) exercises on inhibitory control in children with ADHD is still unclear. Two studies reported that vigorous-intensity aerobic exercise benefits inhibitory control in children with ADHD. Specifically, Gawrilow et al. (2016) found that children with ADHD exhibited better inhibitory control following a single bout of vigorous-intensity aerobic exercise. Similarly, Silva et al. (2015) found that children with ADHD who underwent vigorous-intensity exercise workouts demonstrated identical levels of attention as their typically developing peers, who received no exercise intervention. However, findings from these two studies are confounded by the use of between-subjects design with no pre-test measures, in which pre-existing differences between intervention groups cannot be assessed. In addition, one study investigating the effects of high-intensity interval exercise found a positive effect on sustained attention, but findings are confounded by unequal durations between the exercise and non-active control condition (Medina et al., 2010). Another study employing the same exercise mode and cognitive task found no effect of high-intensity interval exercise on sustained attention, but in this case, the results are confounded by individual differences in medication use (Mahon et al., 2013).

Moreover, although no previous study investigated the differential effects across low, moderate, and vigorous-intensity aerobic exercises on inhibitory control in children with ADHD, a meta-analysis of individuals without ADHD indicated that high- and moderate-intensity exercises, but not light-intensity exercise, benefits cognitive function in adults over 50 years old (Northey et al., 2018). In contrast, another meta-analysis found that the beneficial effect of vigorous-intensity exercise and high-intensity interval training were larger than moderate-intensity exercise (Oberste et al., 2019). In addition, a systematic review found a beneficial effect of low-intensity exercise on cognitive performance (i.e., word fluency) in older adults (Tse et al., 2015). An empirical study further indicated that young adults had better inhibitory control performance following a single bout of low-intensity aerobic exercise relative to following a control condition (Byun et al., 2014). These findings, altogether, suggest that exercise intensity is an important factor when assessing cognitive performance in individuals without ADHD and that there may be a dose-response effect of acute aerobic exercise intensity on inhibitory control in children with ADHD.

The differential effects of low, moderate, and vigorous-intensity aerobic exercises on inhibitory control may be accounted for, at least in part, by fluctuations in cortical arousal. Davey (1973) postulates that moderate-intensity aerobic exercise results in an optimal level of cortical arousal, which, in turn, facilitates cognitive performance, whereas low- and vigorous-intensity exercise may lead to suboptimal and excessive cortical arousal, thereby compromising cognitive performance. This inverted-*U* shape relation has been verified in non-clinical, typically developed populations (Kamijo et al., 2004a; Chang and Etnier, 2009; Chang et al., 2011), but remain underexplored in children with ADHD. Hypoarousal has been consistently reported in children with ADHD (Satterfield et al., 1974; Barry et al., 2003; Rowe et al., 2005). The optimal stimulation (OS) model suggested that the state of arousal of the central nervous system (CNS) drives subsequent response and behavior (Zentall and Zentall, 1983). According to the OS model, hypoarousal in children with ADHD, probably results from deviated maturation/dysfunction in the prefrontal cortex (Strauß et al., 2018; Zhang et al., 2018), is closely related to inattention and hyperactivity (Rapport et al., 2009). Given the modulatory effect of acute exercise on arousal (Kamijo et al., 2004a; Chang et al., 2011), it is reasonable to postulate that better cognitive performance following acute exercise may be mediated by an upregulation of cortical arousal.

To measure cortical arousal, the current study used alpha oscillations from electroencephalography (EEG). Alpha power, or the amplitude of brainwaves between 8 and 13 Hz frequency band, has been shown to be inversely correlated with mental activity. Studies also show a relationship between reduced alpha power (i.e., EEG desynchrony) and increased vigilance, alertness, and attention (see Oken et al., 2006 for a comprehensive review). Furthermore, attenuation of alpha oscillation is inversely associated with fMRI blood-oxygen-level dependent (BOLD) cortical response (Laufs et al., 2006) and is closely related to another standardized measure of arousal, skin conductance, with reduced alpha activity relates to increased skin conductance (Barry et al., 2005). These data suggest that reduction in alpha power is associated with increased cortical arousal, but despite this relation, alpha oscillation has not been broadly utilized in studying the relationship between exercise, arousal, and cognition in children with ADHD.

The Ericksen flanker task has been extensively used to measure inhibitory control in children with ADHD (Mullane et al., 2009). It involves participants being instructed to identify a target stimulus defined by its directionality (i.e., the directionality of an arrow) while ignoring other distracting stimuli that flanking the target stimulus. In general, children with ADHD display longer RT (Liu et al., 2020) and lower response accuracy (Johnstone and Galletta, 2013) compared with typically developing children during the flanker task.

In addition to behavioral indices, event-related brain potentials (ERPs) from EEG affords insights into the specific cognitive processes underpinning inhibitory control (Luck, 2014). In particular, the amplitude and latency of the P3-ERP have been demonstrated to represent the allocation of attention resources and speed of stimulus processing/evaluation, respectively (Polich, 2007). Pontifex et al. (2013) reported that, during a flanker task, children with ADHD displayed larger P3 amplitudes following a 20 min bout of moderate-intensity aerobic exercise relative to the following reading. Similarly, Ludyga et al. (2017) found that in children with ADHD, there were increased P3 amplitudes during a flanker task following a single bout of 20 min moderate-intensity cycling compared with following a seated rest condition. While increases in P3 amplitude may account for improved inhibitory control following acute bouts of moderate-intensity aerobic exercise in children with ADHD, to date we have limited understanding of the effects of low- and vigorous-intensity exercises on P3 amplitudes, or of the differential effects across low, moderate, and vigorous aerobic exercises on P3 amplitude. Given that modulations in P3 amplitude are closely related to cortical arousal changes (Kamijo et al., 2004b), it could be speculated that different exercise intensities would differentially modulate P3 amplitude.

The purpose of this current study was to investigate whether the effects of acute exercise on inhibitory control would be moderated by different exercise intensities. With regards to cortical arousal fluctuation, it was hypothesized that arousal would increase with exercise intensity, as reflected by decreased alpha power. Moderate-intensity exercise would result in an optimal state of cortical arousal, whereas low-and vigorous-intensity exercises would result in a suboptimal state of cortical arousal. Similarly, it was hypothesized that moderate-intensity exercise would result in better inhibitory control performance, which could be reflected by shorter RT and/or higher response accuracy, as well as larger P3 amplitude relative to low-and vigorous-intensities.

Deficits in inhibitory control and its neuroelectric outcomes are core neurocognitive deficits in children with ADHD (McAuley et al., 2014) and have negative impacts on various cognitive domains (Miyake et al., 2000). As such, an examination to whether there is a dose-response relation between acute aerobic exercise intensity and inhibitory control, as well as an understanding of possible underlying mechanisms that may drive acute exercise effects, affords a better understanding of the optimal acute exercise protocols to aid inhibitory control.

## Materials and Methods

### Participants

Thirty-two children from local elementary schools were screened following referrals from special education teachers and parents. This sample size was determined based on an a priori power analysis using G^*^Power 3.1 (Faul et al., 2007). Assuming an effect size ($\eta_{\text{p}}^{2}$ = 0.33) reported in previous studies (Pontifex et al., 2013; Ludyga et al., 2017), a sample size of 24 participants was satisfactory to reach a power of 0.80 at an alpha level of 0.05. Although the power analysis indicated that a sample of 24 children was satisfactory, we recruited 32 participants to compensate for any dropouts, incompliance, or bad data. The inclusion criteria were as follows: (1) the child has had been diagnosed with ADHD by a certified psychiatrist by following the diagnosis criteria by the DSM-IV (Diagnostic and Statistical Manual of Mental Disorders, Fourth Edition); (2) aged between 7 and 12 years; and (3) no history of brain injury or neurological conditions, such as epileptic seizures, serious head injuries, or periods of unconsciousness.

For supplementary assessments to further verify symptoms of ADHD, the Chinese version of the ADHD test originally developed by Gilliam (1995) (ADHD-T) using traditional characters (Cheng, 2008) was completed by the parents to assess the severity of ADHD symptoms in each child. Inter-rater reliability and test-retest reliability of ADHD-T was above 0.9 and 0.8–0.92, respectively (Cheng, 2008). Those with ADHD typically score in the range of 46–155 in this test. Participants in the present study had a mean score of 105.72 ± 12.73, indicating a moderate severity of ADHD symptoms (Gilliam, 1995). In addition, the parent-rated Child Behavior Checklist (CBCL), which has been used as diagnostic tools to identify clinical disorders and quantify the severity of psychopathology in children and adolescents (Chang et al., 2016). The present study adapted the attention problem scale, a subscale of the CBCL, to index ADHD symptom levels. The children in the present study had *t*-score of 72.75 ± 9 where a *t*-value of 60 is considered as the cut-off score for the clinical range (Aebi et al., 2010).

Of those recruited, seven participants were excluded from the present study. Four of them failed to complete the whole experiment and three had <50% accuracy in the task due to (a) they did not understand the instructions, (b) felt bored during the task, or (c) displayed disruptive behavior. The criterion of 50% response accuracy was utilized by a previous study that also focused on children with ADHD (Yu et al., 2020). Of the remaining 25 children (mean age = 10.54, SD = 1.17), 16 were taking stimulant medications such as Ritalin (*n* = 6), Concerta (*n* = 9), and Strattera (*n* = 1), and they were instructed to abstain from medications for at least 24 h prior to each visit (Johnstone et al., 2009). Of the 25 children, one child was also diagnosed with autism spectrum disorder and the other 24 children did not have any co-morbidities. Before starting experimental sessions, written informed consent and assent forms approved by the Institutional Review Board at National Taiwan University were completed by the parents and children, respectively. The demographic characteristics of participants are summarized in Table 1.

**Table 1:** Demographic characteristics of participants.

**Characteristics**	**ADHD (*N* = 25)**
Gender (male/female)	23/2
Age (yr)	10.54 (1.17)
Height (cm)	146.60 (10.25)
Weight (kg)	42.40 (11.53)
BMI	19.37 (2.78)
Subtype of ADHD	***N*** **(%)**
Inattentive	11 (44)
Impulsive/hyper	0 (0)
Combined	14 (56)
Taking medication	16 (64)

*All data presented as mean (SD) unless otherwise noted*.

### Flanker Task

The computerized Eriksen flanker task (Eriksen and Eriksen, 1974) used in this study incorporated two types of stimulus: “congruent,” where all arrows point in the same direction (< < < < < or > > > > >) and “incongruent,” where the central target arrow points in a different direction to the adjacent arrows (< < > < < or > > < > >). In addition, cognitive loads were varied by instructing participants to complete the task under two different trial conditions, “compatible” and “incompatible.” In the “compatible” condition, target arrows pointing left or right required a response using the fingers of the corresponding left or right side. Thus, in a “compatible” trail, if the target arrow was facing left, participants were instructed to press the A button (located on the left side of the keyboard) with their left index finger. If the target faced right, participants were instructed to press the L key (on the right side of the keyboard) with their right index finger. An “incompatible” trial required participants to press the A key when the target arrow faced right and the L key when it faced left. Cognitive loads were increased in the incompatible condition since participants were not only required to exhibit inhibitory control, but also cognitive flexibility and working memory (Chaddock et al., 2012). Varying congruency and compatibility produced four experimental conditions: Compatible-Congruent (CC), Compatible-Incongruent (CI), Incompatible-Congruent (IC), and Incompatible-Incongruent (II), with CC and CI trials preceding IC and II trials. The “compatible” and “incompatible” tasks each consisted of three blocks. Each block was made up of 60 trials with an equal number of congruent and incongruent stimuli. Each trial began with a central fixation cross (+) lasting 1,000 ms, followed by the target stimulus for 200 ms displayed at the center of the computer screen. The next trial began after a button-press response was made or at the end of the response window of 1,200 ms from the time of the presentation of a target stimulus. Participants performed all blocks in a single session with a 1 min break between each block. The total duration of the flanker task was ~30 min.

### Exercise Manipulation Check

Heart rate was recorded with a Polar HR monitor before the exercise session (pre-HR), and then every 30 s during the main stage (20 min) of 30 min session of exercise (exercise HR). After exercise, HR (post-HR) was recorded during a resting period prior to performing the flanker task. During the main exercise stage, ratings of perceived exertion (RPE) were also taken every 2 min to estimate perceptions of individuals of the physical demands of the exercise (Borg, 1982).

### Procedures

Each participant was asked to visit the laboratory at the same time of day on four separate occasions, with at least 3 days apart between each visit. On Day 1, parents and their children were given a brief introduction to the study by a trained experimenter. Upon arrival, parents were instructed to complete the paperwork (an informed consent form, the medication status of the child, and psychological conditions), and the child was instructed to administer with a battery of physical fitness and motor skill tests (refer to related test Hsieh et al., 2017), which was not reported in the current study.

From Day 2 to 4, participants underwent the low, moderate, and vigorous exercise sessions in a counterbalanced order. Given that a growing body of evidence has indicated a positive effect of moderate-intensity exercise on inhibitory control in children, including children with ADHD (Suarez-Manzano et al., 2018), we did not include a non-exercise control session in the current study. This decision was made in order to avoid practice or familiarization effect, as well as to keep children away from becoming bored with the study protocol.

In each session, before engaging in exercise (Pre-EX), participants were first seated comfortably on a chair and fitted with an electrode cap and a resting EEG was recorded for 2 min with open (O) eyes of the participant (O) and fixated upon a cross on the computer screen. Another 2 min of recording was then conducted with closed (C) eyes of the participants. This was then repeated (i.e., the order of recording was OCOC) one time. A resting HR measure was also taken during this time for later calculation of target HR. This was followed by a 30-min exercise session consisting of 5 min of warm-up, 20 min of exercise, and then a 5-min cool-down period. The same protocol (i.e., exercise with a cap on) has been utilized by several previous studies (Kamijo et al., 2004b; Pontifex et al., 2015; Hung et al., 2016; Ludyga et al., 2017; Kao et al., 2018; McGowan et al., 2019; Yu et al., 2020).

The intervention consisted of low, moderate, or vigorous exercises defined as running on a treadmill with a target HR of 30, 50–60, and 70–80% of individual HR reserve (HRR), respectively. These three exercise intensities were adapted from the classification developed by Norton et al. (2010), where 20–40% HRR, 40–60% HRR, and 60–85% HRR represented light, moderate, and vigorous-intensity exercises, respectively, and are aligned with previous studies that also investigated the effects of aerobic exercise on inhibitory control in children with ADHD (Chang et al., 2012; Chuang et al., 2015; Yu et al., 2020). HRR was calculated as maximal HR minus resting HR (Karvonen et al., 1957), where the maximal HR was estimated using the formula “206.9 – (0.67 × age)” (Gellish, 2007). The target HR was calculated as follows: Target HR = {[(maximal HR– resting HR) × percentage intensity desired] + resting HR}.

Following the completion of the interventions, another resting EEG with eyes open was recorded for 2 min (After-EX). Participants were then provided with an explanation of the flanker task and allowed 10 practice trials. The compatible flanker task was performed within about 12 min after exercise, and there was no significant difference in this time interval between the various treatment conditions [*F*_(2, 48)_ = 1.78, *p* = 0.18, and $\eta_{p}^{2}$ = 0.07] (average time interval: low intensity M = 13.04 ± 4.90 min; moderate intensity M = 11.33 ± 1.94 min; and vigorous intensity M = 12.94 ± 4.20 min). This was followed by the incompatible task conditions (IC and II), which occurred around 30 min after exercise, and there was also no significant time difference between treatment conditions [*F*_(2, 48)_ = 1.63, *p* = 0.21, and $\eta_{p}^{2}$ = 0.06] (average interval time: low intensity M = 31.92 ± 6.33 min; moderate intensity M = 31.03 ± 4.46 min; and vigorous intensity M = 29.76 ± 4.52 min). Before the incompatible condition of the flanker task, resting EEG was recorded again for 2 min with eyes open and for 2 min with eyes closed (In-task). Note, the present study analyzed only the data recorded with eyes open to compare it with the resting EEG after the acute exercise which was only recorded with eyes open. Upon completion of all sessions, parents of the participants received US$65 remuneration for their participation in the experiment as well as a brief individual report on the performance of their child.

### Electroencephalography Recording

Electroencephalographic activity was measured at 30 sites using a NeuroScan Quik-Cap (Neuro, Inc., Charlotte, NC, USA) according to a modified 10–20 system (Jasper, 1958). Ongoing EEG activity was referenced to the average of the mastoid (A1, A2), with AFz serving as the ground electrode. Electrooculographic (EOG) activity, potential responses generated by eye movements, was measured using electrodes placed at the outer canthus of each eye and above and below the left orbit. Electrode impedances were kept below 10 kΩ. EEG signals were recorded and sampled at 500 Hz using a DC−100 Hz filter and a 60 Hz notch filter.

Considering the possibility of electrode bridges and non-brain derived artifacts when participants exercised with the EEG caps on, we have several procedures to mitigate these issues. First, before data collection, we adjusted the cap position if there was any shifting caused by movements. Given that sweat may continue after exercising, the room temperature was controlled under 26°C to avoid excess perspiration. We also do not overfill the gel into each electrode during cap preparation to avoid potential bridging between electrodes, especially after exercise with sweat. In addition, the impedance was kept below 10 kΩ for all electrodes. Research indicated that valid EEG signals could be recorded with impedance <40 kΩ (Ferree et al., 2001) and the conductance of sweat could be minimized with an impedance closed to 5 kΩ (Picton and Hillyard, 1972). Second, during the data processing, we did filtering (filtering out non-brain derived signals or artifacts), baseline correction (could mitigate data drifting caused by sweat), and artifact rejection (discarded epochs that contain excess amplitude caused by artifacts), all these protocols could address artifacts. Lastly, before averaging the data, we had a visual inspection of all available epochs to see if there were epochs with bad data. Epochs with bad data would be discarded. We compared the number of trials included for averaged data across three exercise intensity conditions to see whether the trials included for finalized data differ as a function of intensity. In general, greater artifacts would result in a greater number of trials being rejected during reduction and artifact rejection. The mean numbers of accepted congruent/incongruent trials for the compatible task and incompatible tasks following low-intensity condition (71.60 ± 8.21/68.52 ± 7.08; 69.68 ± 9.72/69.32 ± 10.24), moderate-intensity condition (75.20 ± 7.40/68.48 ± 8.81; 70.08 ± 7.66/70.04 ± 8.92), and vigorous-intensity condition (71.24 ± 9.61/65.88 ± 8.47; 66.72 ± 10.67/66.88.5 ± 11.58) does not differ from each other (*ps* > 0.05). This suggested that data quality across three exercise conditions could be consistent and homogenous.

### Data Reduction

#### Behavioral Performance

The median absolute deviation method suggested by Leys et al. (2013) was used to detect outliers in behavioral performance (RT and accuracy) with the deviation threshold set to 2.5, which is considered moderately conservative (Miller, 1991). Behavioral performance was measured in terms of RT for correct responses as well as accuracy (Acc) (the percentage of trials that successfully responded to target stimulus) of congruent and incongruent conditions for compatible and incompatible tasks.

#### Event-Related Potentials and Resting EEG

**Figure 1:**
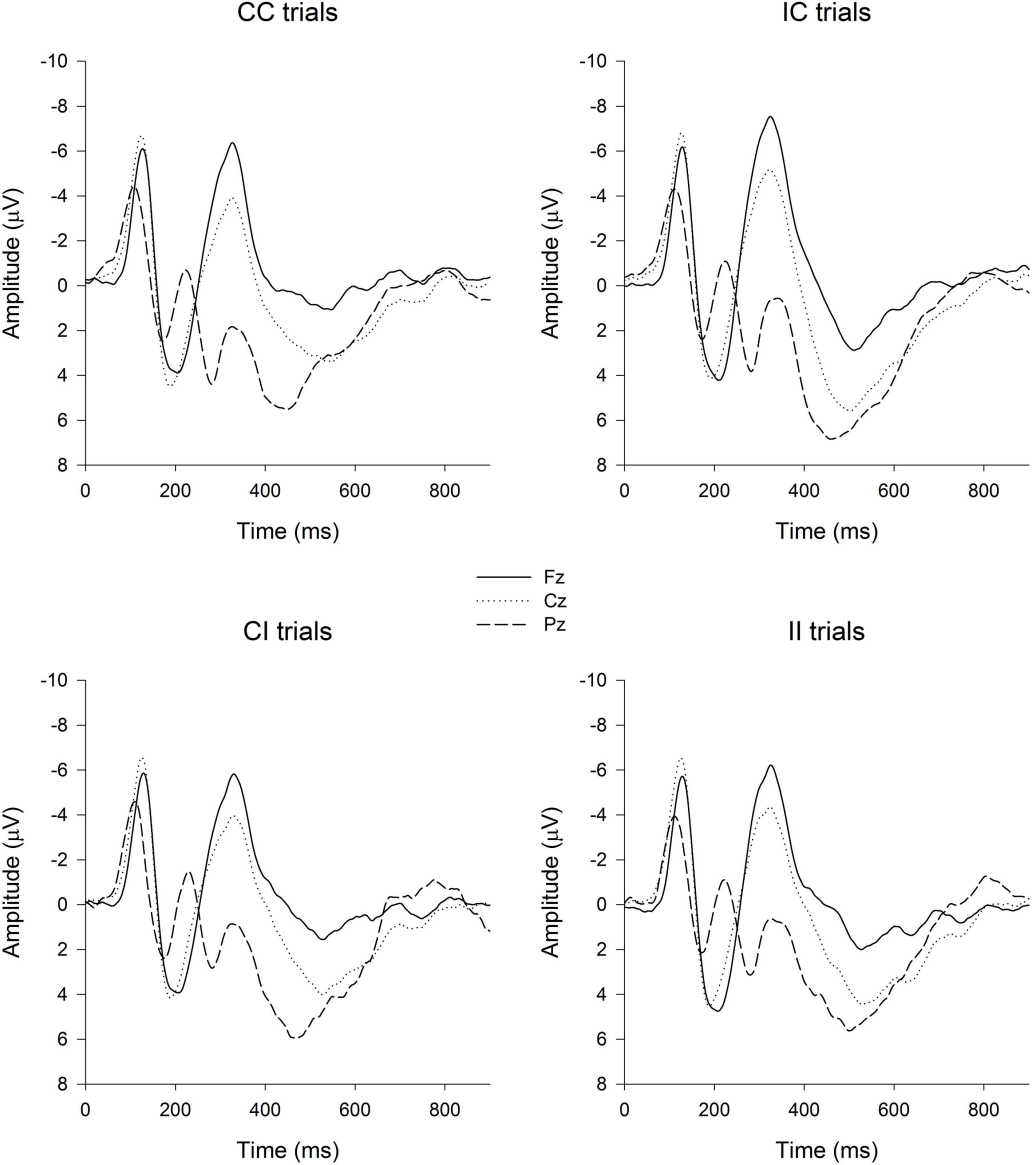
Grand-averaged waveforms in CI, CC, IC, and II, respectively. CC, compatible congruent; CI, compatible incongruent; IC, incompatible congruent; II, incompatible incongruent.

**Figure 2:**
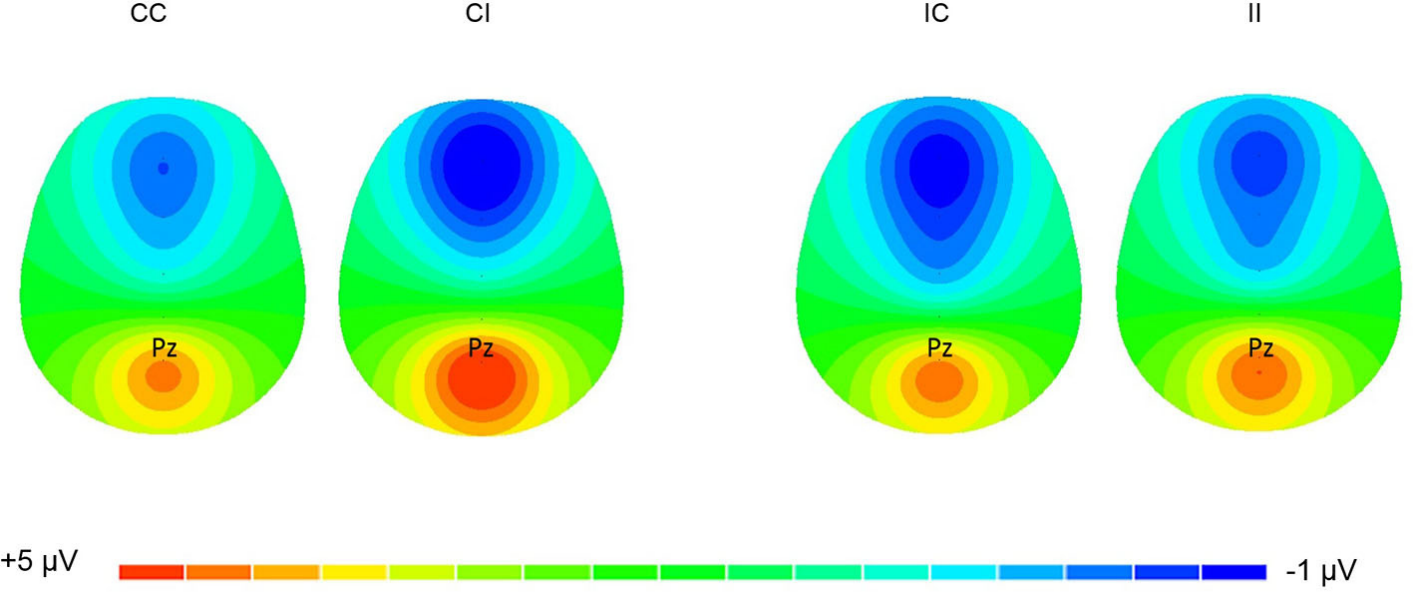
Topographic distribution of P3 (400–550 ms). The topographic distribution of P3 amplitude (spectrum: blue to red) is illustrated in CI, CC, IC, and II, respectively. CC, compatible congruent; CI, compatible incongruent; IC, incompatible congruent; II, incompatible incongruent.

The EEG/ERP data were processed offline with Scan 4.5 software (Compumedics USA, Ltd., El Paso, TX, USA). EOG activity was corrected using the algorithm developed by Semlitsch et al. (1986) on both ERP and resting EEG data. For ERP recording, epochs were defined as 100 ms prior to the stimulus to 1,500 ms after the stimulus. The baseline was defined as the mean amplitude of the 100 ms pre-stimulus interval. The data were filtered using a 30 Hz low-pass cutoff (12 dB/octave), and ERP trials with an amplitude outside the range of ± 100 μV were excluded. Only correct responses were averaged. The mean number of trials for each condition was 72.68 ± 8.53 (CC), 67.63 ± 8.14 (IC), 68.83 ± 9.43 (IC), and 68.75 ± 10.56 (II). Peak detection was only performed at the Pz electrode from the grand-averaged waveforms (Figure 1), and this electrode selection was made by referring to the scalp distribution of P3 (Figure 2) as well as previous works (Polich, 2007). P3 latency was defined as the latency of the positive peak between the 300–800 ms time window after stimulus onset. P3 amplitude was defined as the mean amplitude within a 50 ms interval surrounding the positive peak (Pontifex et al., 2013).

For resting alpha oscillations, epochs were defined as 2,000 ms. The baseline was defined as the mean amplitude of the entire interval. The data were filtered using a 1–30 Hz band-pass cutoff (12 dB/octave), and EEG trials with an amplitude outside the range of ±100 μV were excluded. The mean number of trials for the time point of vigorous-intensity exercise was 55.82 ± 9.85 (Pre-EX), 58.32 ± 7.78 (After-EX), and 59.64 ± 5.91 (In-Task). For the time point of the moderate-intensity exercise, the mean number of trials was 56.75 ± 10.78 (Pre-EX), 57.04 ± 14.04 (After-EX), and 56.43 ± 13.74 (In-Task). The mean number of trials for time point of low-intensity exercise was 57.50 ± 9.23 (Pre-EX), 58.43 ± 10.58 (After-EX), and 60.36 ± 6.89 (In-Task). The cleaned EEG data were Fast Fourier Transformed and subsequently log-transformed. The power value for alpha (8–13 Hz) at Fz, Cz, and Pz were calculated using fixed frequency bands (Kamijo et al., 2004a).

### Statistical Analysis

A repeated-measure ANOVA was utilized to assess the effect of exercise intensity (i.e., low, moderate, and vigorous exercise) on RPE. The HR changes were further examined by a 3 (*Time*: pre-HR, exercise-HR, and post-HR) × 3 (*Intensity*: low exercise, moderate exercise, and vigorous exercise) repeated-measure ANOVA (RM ANOVA). Additionally, a 3 (*Intensity*) × 3 (*Time*: Pre-EX, After-EX, In-task) × 3 (*Electrode*: Fz, Cz, Pz) RM ANOVAs were used to test fluctuations in alpha power. To test the effects of exercise intensity on behavioral performance (i.e., RT and response accuracy), separate 3 (*Intensity*) × 2 (*Compatibility*: compatible and incompatible) × 2 (*Congruency*: congruent and incongruent) RM ANOVAs were performed. Individual (*Intensity*) × 2 (*Compatibility*) × 2 (*Congruency*) RM ANOVAs were conducted to test the effects of exercise intensity on P3 amplitude and latency. The Greenhouse-Geisser correction was used whenever the sphericity assumption was violated. Fisher's Protected Least Significant Difference (LSD) tests for multiple comparisons were performed to follow up significant effects, an approach considered appropriate when the level of a factor is <3 (Baguley, 2012). A family-wise alpha of 0.05 was utilized, and effect sizes of ANOVAs were calculated using partial eta-squared ($\eta_{p}^{2}$) statistics, and effect sizes for *post-hoc* of t-tests were reported by Cohen's *dz* (for within-subject designs).

To further investigate whether changes in ERP and alpha power measures related to changes in behavioral outcomes, Pearson product-moment correlations were performed wherever significant exercise effects were detected. Data of alpha power were obtained by subtracting Pre-EX from After-EX as well as subtracting Pre-EX from In-Task to see if the change of alpha power is associated with cognitive performance.

## Results

### Manipulation Check for Exercise Intensity

Descriptive data for RPE, HR, and alpha values are summarized in Table 2. As expected, there was a significant increase in RPE with exercise intensity [*F*_(2, 48)_ = 48.76, *p* < 0.001, and $\eta_{p}^{2}$ = 0.67]. An analysis of HR revealed a significant *Intensity* × *Time* interaction [*F*_(4, 96)_ = 138.29, *p* < 0.001, and $\eta_{p}^{2}$ = 0.85]. Follow-up simple main effect analyses found that while exercise-HR and post-HR increased with exercise intensity, there were no differences between intensity in pre-HR.

**Table 2:** Descriptive data for exercise manipulation check and arousal level.

**Variable**	**Low**	**Moderate**	**Vigorous**	***P***	**Direction**
	**M (SD)**	**M (SD)**	**M (SD)**		
RPE	10.32 (1.94)	13.03 (2.44)	15.06 (2.38)	<0.001	L < M < V
HR-pre (bpm)	81.18 (8.97)	83.22 (25.62)	83.64 (7.23)	n.s	
HR-avg. (bpm)	118.07 (9.54)	144.37 (5.99)	163.78 (5.28)	<0.001	L < M < V
HR-post (bpm)	82.68 (11.45)	90.74 (12.03)	97.86 (8.54)	<0.001	L < M < V
Alpha (Ln μV^2^)					
Pre-EX^*^	1.68 (0.56)	1.58 (0.54)	1.59 (0.63)	n.s	
After-EX^*^	1.85 (0.58)	1.69 (0.55)	1.52 (0.60)	<0.001	L>M>V
In-Task^*^	1.73 (0.53)	1.62 (0.58)	1.41 (0.56)	<0.001	L>M>V

* *Data was collapsed across electrode sites*.

### Behavioral Performance

For RT, there was a significant *Intensity* × *Compatibility* interaction [*F*_(2, 48)_ = 4.28, *p* = 0.019, and $\eta_{p}^{2}$ = 0.15]. Follow-up analyses indicated longer RT in the incompatible condition regardless of congruency after a session of vigorous-intensity exercise compared to moderate exercise and low exercise sessions, with no difference between the latter two (see Table 3).

**Table 3:** Task performance by exercise intensity during compatible and incompatible tasks.

**Variable**	**Low**	**Moderate**	**Vigorous**	***P***	**Direction**
	**M (SD)**	**M (SD)**	**M (SD)**		
**Compatible**					
RT (ms)	571.09 (83.77)	577.33 (81.02)	566.32 (72.27)	n.s	
Acc (%)	92.37 (3.81)	92.77 (5.58)	92.16 (5.26)	n.s	
**Incompatible**					
RT (ms)	573.44 (83.05)	576.96 (77.08)	601.35 (93.45)	0.03	L&M < H
Acc (%)	91.73 (4.53)	90.66 (5.36)	89.96 (4.60)	n.s.	

*RT, reaction time; Acc, accuracy*.

For response accuracy, analysis revealed a significant *Compatibility* × *Congruency* interaction [*F*_(2, 48)_ = 52.62, *p* < 0.001, and $\eta_{p}^{2}$ = 0.69). Follow-up analyses indicated higher response accuracy in CC trials (M = 95.3 %, SD = 3.3%) compared with CI trials (M = 89.6 %, SD = 4.8%) as well as compared with IC trials (M = 91.2%, SD = 2.9%).

### Neuroelectric Measures

Table 4 summarizes the data relating to P3 latency and amplitude. In terms of P3 latency, the analysis found significant *Intensity* × *Compatibility* × *Congruency* interactions [*F*_(2, 48)_ = 4.80, *p* = 0.013, and $\eta_{p}^{2}$ = 0.17]. Further decomposition found a significant *Intensity* × *Congruency* interaction in the incompatible condition [*F*_(2, 48)_ = 4.73, *p* = 0.016, and $\eta_{p}^{2}$ = 0.17), with longer P3 latencies during the II trials compared with IC trials following vigorous (*p* = 0.04) and low intensity exercises (*p* = 0.02) but not following moderate-intensity exercise. In addition, decomposition found a significant *Compatibility* × *Congruency* interaction following moderate-intensity exercise [*F*_(1, 24)_ = 7.12, *p* = 0.013, and $\eta_{p}^{2}$ = 0.23], with CC trials having shorter latency compared with CI trials and IC trials. No congruency difference was found in the incompatible condition and no compatibility difference was found in the incongruent condition. No *Compatibility* × *Congruency* interaction was observed following vigorous-and low-intensity exercises.

**Table 4:** Mean (SD) of event-related brain potential (ERP) data.

**Variable**	**P3L**				**P3A**			
	**Exercise intensity**				**Exercise intensity**			
	**Low**	**Moderate**	**Vigorous**		**Low**	**Moderate**	**Vigorous**	
	**M (SD)**	**M (SD)**	**M (SD)**	***p***	**M (SD)**	**M (SD)**	**M (SD)**	***p***
**Compatible**								
Congruent	468.72 (68.58)	448.32 (68.23)	452.16 (57.19)	n.s.	6.37 (2.78)	6.22 (2.64)	6.29 (3.49)	n.s.
Incongruent	474.40 (63.03)	478.96 (59.99)	476.40 (59.25)	n.s.	7.71 (2.85)	7.85 (3.25)	7.42 (3.93)	n.s.
**Incompatible**								
Congruent	460.72 (73.76)	486.56 (70.45)	466.75 (62.68)	n.s.	5.42 (3.21)	5.60 (3.31)	5.73 (2.47)	n.s.
Incongruent	518.56 (74.88)	477.68 (76.59)	482.83 (55.21)	n.s.	6.43 (3.62)	6.23 (3.89)	5.96 (2.81)	n.s.

*A, amplitude; L, latency*.

Regarding P3 amplitude, a significant *Compatibility* × *Congruency* interactions [*F*_(1, 24)_ = 6.07, *p* = 0.021 and $\eta_{p}^{2}$ = 0.11] was found. Follow-up analyses indicated larger P3 amplitudes in CI trials compared to CC trials, and II trials compared with IC trials (*p'*s < 0.04). In addition, there were larger P3 amplitudes in CI trials compared with II trials (*p* < 0.001). No intensity associated main effect or interaction effects was observed.

### Arousal Level

**Figure 3:**
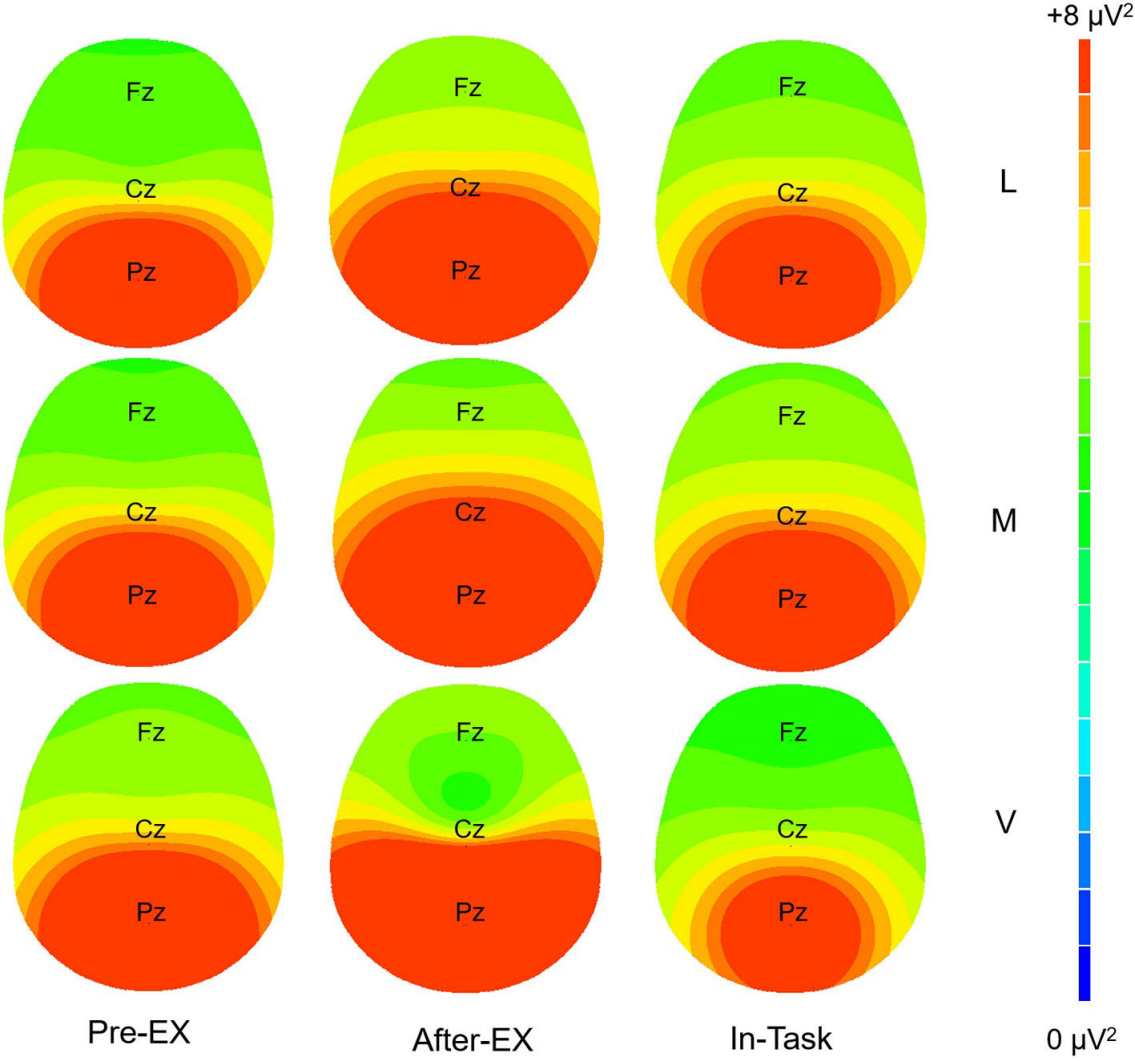
Topographic distribution of alpha power. The topographic distribution of alpha power (spectrum: blue to red) is illustrated for three intensity exercises and time, respectively. L, low-intensity exercise; M, moderate-intensity exercise; V, vigorous-intensity exercise.

An analysis of alpha power found a significant interaction of Intensity × Time [*F*_(4, 96)_ = 6.40, *p* < 0.001, and $\eta_{p}^{2}$ = 0.21). Decomposition of Intensity × Time interaction compared intensity within each time and found significant effects after exercise (After-EX) and during the flanker task (In-Task). Follow-up analyses found mean alpha power decreased with exercise intensity for both After-EX and In-Task, indicating that there was a linear increase of cortical arousal with the increase of exercise intensity 10 min or around 30 min after exercise (see Table 2). Comparing different times within each intensity, the mean alpha power decreased during In-Task relative to Pre-EX following vigorous-intensity exercise, indicating that the arousal level was sustained higher for a while after exercise cessation. However, the opposite trend was found following low-intensity exercise where the mean alpha power increased in After-EX relative to Pre-EX (Cohen's *dz* = 0.98) as well as In-Task (Cohen's *dz* = 0,54), indicating that the arousal level was at its lowest level around 10 min after exercise cessation. A similar trend was also found After-EX relative to Pre-EX (Cohen's *dz* = 0.48) following moderate-intensity exercise, although this was not statistically significant (*p* = 0.06) (Figure 3).

### Bivariate Correlations

Given the significant exercise intensity effects were detected on incompatible RT across congruency trials and alpha power change, bivariate correlations were separately calculated between incompatible RT and alpha power change under each exercise intensity condition. Results revealed a negative correlation of alpha power change from baseline to After-EX with incompatible RT (*r* = −0.41 and *p* = 0.45) following the moderate-intensity exercise. No other significant correlation between alpha power change and incompatible RT was found under low-intensity condition (*r's* = −0.051–0.18 and *p's* > 0.80) or vigorous-intensity condition (*r's* = 0.005–0.040 and *p's* > 0.86).

## Discussion

The purpose of this study was to investigate whether the effects of acute aerobic exercise on inhibitory control in children with ADHD are moderated by exercise intensity and to examine the role of arousal as a potential mechanism for effects of exercise intensity on inhibitory control. The results indicated no difference across exercise intensities in behavioral outcomes during the compatible condition. On the other hand, results also showed longer RT in the incompatible condition after a 20 min bout of vigorous-intensity exercise, compared with both low and moderate-intensity exercises. Regarding P3, there is an interference effect following low- and vigorous-intensity exercises, which was not seen in the incompatible task after moderate-intensity exercise. Additionally, cortical arousal increased with greater exercise intensity, as reflected by decreased alpha power. Similarly, higher cortical arousal was sustained following vigorous-intensity exercise. In contrast, cortical arousal was decreased following low- and moderate-intensity exercises, though in the latter condition the relationship fell short of statistical significance. Lastly, a negative correlation was found between changes in arousal level from Pre-Ex to After-Ex and incompatible RT following moderate-intensity exercise. Since we did not include a control condition, caution must be exercised in interpreting our findings. However, despite these limitations, the present study does provide preliminary evidence for a dose-related effect of aerobic exercise intensity on behavioral and neuroelectric outcomes of inhibitory control in children with ADHD. Our findings are among the first to demonstrate the moderating role of aerobic exercise intensity on the association of acute aerobic exercise with inhibitory control and cortical arousal in children with ADHD.

Results indicated shorter RT in the incompatible condition following low- and moderate-intensity exercises relative to vigorous-intensity exercise, with no difference between the former two. A lack of difference between low- and moderate-intensity exercises was unexpected and inconsistent with the inverted-U hypothesis which predicts that low-intensity exercise would result in poorer cognitive performance relative to moderate-intensity exercise due to a suboptimal level of cortical arousal. A parallel finding was reported in a pharmaceutical study, which found that both low and moderate doses of methylphenidate (MPH) had identical positive effects on academic performance (Evans et al., 2001). The study findings specific to light-intensity exercise have practical implications since children with ADHD have been found to have a higher risk of obesity (Cortese et al., 2013). Research has recommended that children with overweight or obesity may have greater adherence and compliance to light-intensity exercise such as brisk walking, as compared with more vigorous exercise. Further, a study also suggests that with higher adherence and better compliance, light exercise may manifest equally beneficial effects compared with exercise with higher intensities in the longer term (Bar-Or, 2000). As such, low-intensity aerobic exercise could be an alternate physical activity choice for children with ADHD, especially children that are less active and are affected by overweight or obesity (Tse et al., 2015).

The finding that vigorous-intensity exercise resulted in poorer cognitive performance relative to moderate-intensity exercise is in line with most previous studies in typically developing children (Duncan and Johnson, 2014) and adults (Kamijo et al., 2004a,b; Budde et al., 2010; Chang et al., 2011). The poorer cognitive performance following vigorous exercise may be explained by neurobiological mechanisms. Exercise results in the secretion of norepinephrine (NE) and dopamine (DA) (Kitaoka et al., 2010; Goekint et al., 2012; see McMorris and Hale, 2015, for review), which stimulates neural signaling (Deutch and Roth, 1990) and dampens neural noise (Finlay et al., 1995), respectively, thereby increases vigilance and arousal. However, excessive secretion of NE and DA, such as that induced by vigorous exercise, impairs neural signaling and increases neural noise despite arousal being high. This dose-related relation between dopamine and cognitive performance is further supported by neuro-pharmacological findings. For example, MPH and atomoxetine (ATM), two common dopaminergic stimulants for children with ADHD, in general, have negative impacts on cognitive performance with excessive doses (Gamo et al., 2010; Arnsten and Pliszka, 2011).

These findings were inconsistent with previous studies that showed beneficial effects of vigorous-intensity exercise on cognitive performance in children with ADHD (Silva et al., 2015; Gawrilow et al., 2016). However, it should be noted that previous studies did not provide any information on HR or RPE to confirm the manipulation of intensity. Therefore, it is not only difficult to assess whether the participants did, indeed, participate in the vigorous-intensity exercise as the authors claimed in their study, but also make comparisons between these studies and this study impossible. Furthermore, participants in previous studies engaged in only 5 min of vigorous-intensity exercise, compared with the current study involving 20 min of exercise. It seems that such a long duration as 20 min of vigorous exercise might result in fatigue which impaired subsequent inhibitory control performance, which was in line with a study in healthy young adults (Kamijo et al., 2004a). Notably, the interpretation of vigorous-intensity needs to be cautious because we cannot indicate the directionality (e.g., impaired following vigorous exercise or improved following low and moderate exercise) of exercise effects without a control condition.

On the other hand, we did not observe differences across interventions in behavioral measures during the compatible task condition. This finding may imply that, for task components requiring lower cognitive loads, exercise intensity does not moderate task performance in children with ADHD. This finding supports one previous study that reported improved inhibitory control performance following 20 min bouts of aerobic exercise regardless of intensity in adults with multiple sclerosis (Sandroff et al., 2016). In summary, our findings suggest that, in children with ADHD, these three exercise intensities may have similar effects on tasks involving lower cognitive loads, whereas vigorous-intensity aerobic exercise seems to adversely affect performance on tasks involving high cognitive demand.

Furthermore, we did not observe any effect of exercise intensities on response accuracy during either compatible or incompatible tasks. One review suggests that the effect of acute exercise on accuracy-dependent measures may depend on the cognitive loads of a cognitive task (McMorris and Hale, 2012). For example, the Flanker task may be less cognitively demanding as compared with tasks assessing more complex cognitive operations (e.g., planning, abstract, and reasoning), and, as such, it would be easier for participants to reach a ceiling effect in their performance (Etnier and Chang, 2009). Future studies are encouraged to employ cognitive tasks with different levels of complexity to verify whether the effects of exercise on response accuracy are dependent on the complexity and nature of cognitive tasks.

Regarding the relationship between exercise intensity and arousal, this study found that arousal, as measured by changes in alpha power, increased with exercise intensity and that the highest arousal level, as reflected by greater decreases in alpha power, was associated with relatively sub-optimal performance. This finding partially supports the arousal-performance hypothesis (Cooper, 1973), suggesting that an excessive level of cortical arousal induced by vigorous-intensity aerobic exercise might result in relatively poor behavioral performance. In contrast, there was a moderate increase in alpha power after both low and moderate-intensity aerobic exercises. Our data further indicated a negative correlation between changes in alpha power and RT in incompatible flanker tasks after moderate-intensity exercise, with greater increases in alpha power corresponds to shorter incompatible RT. The results of moderate-intensity aerobic exercise on alpha oscillation align with previous studies that also found increased alpha power after moderate-intensity exercise (Crabbe and Dishman, 2004; Brummer et al., 2011). Further, the negative association between alpha power and RT when engaged in incompatible flanker tasks may imply that a moderate level of cortical arousal is associated with better performance on task components requiring greater inhibitory control after moderate-intensity exercise. Increased alpha power has been found to underpin top-down neuro-inhibition of task-irrelevant information (Janssens et al., 2018). As such, while both low- and moderate-intensity aerobic exercise may result in optimal cortical arousal, moderate-intensity aerobic exercise further enhances inhibitory control *via* improved neuro-inhibition to task-irrelevant information in children with ADHD.

Consistent with previous studies (Hillman et al., 2009; Moore et al., 2013), longer P3 latency and larger P3 amplitude were observed for the incongruent compared with congruent condition, indicating an effective task manipulation so that an examination of modulations in P3-ERP after the different intensities of the acute exercise was possible. It is noteworthy that a congruency effect on P3 latency was observed following low- and vigorous-intensity exercises but not following moderate-intensity exercise. Since larger congruency effect has been considered an indication of inefficient interference control (Mullane et al., 2009), the findings may indicate that moderate-intensity exercise, but not light- or vigorous-intensity exercise, increases interference control and reduces conflicts between task-relevant and irrelevant information at a neurocognitive level.

Several limitations of the current study should be acknowledged. First, given the lack of a pre-test assessment or non-exercise control condition, our data could not verify whether low and moderate aerobic exercise improves inhibitory control. However, the positive influence of acute aerobic exercise on inhibitory control in children with ADHD has been well-documented by several studies employing a non-exercise control session (Chang et al., 2012; Pontifex et al., 2013; Benzing et al., 2018), and it is reasonable to assume that the beneficial effect of acute exercise on inhibitory control reported in those studies would generalize to the present study given the similar exercise protocols used. Second, the classification of vigorous-intensity exercise (i.e., 70–80% of HRR) somewhat overlaps with the moderate intensity in previous studies (50–70% HRR) (Chang et al., 2012; Hung et al., 2016). Such overlap would lead to an underestimation of the differential effects between moderate and vigorous exercise. Despite this, significant differences between these exercise intensities were still observed in measures of HR, RPE, and arousal level. Third, we only recruited children with the “inattentive” and “combined” subtypes of ADHD, and thus, the current finding may not be generalizable to children with other subtypes of ADHD, such as the hyperactive/impulsive subtype. Fourth, almost all the participants were boys, which limit the generalizability of findings to girls. Last but not least, we did not take account the medication use of participants. However, since all participants were asked to refrain from medication for at least 24 h before each visit, any confounding effect from the medication was likely to have been minimized.

## Conclusion

The present study investigated the acute effects of different exercise intensities on inhibitory control in children with ADHD using behavioral indices and P3-ERP. The current findings suggest that children with ADHD may have better inhibitory control following low- and moderate-intensity aerobic exercises relative to vigorous-intensity exercise, and that this may be due to the suppression of distractors by increased alpha power following exercise. It should, however, be noted that findings from this study must be interpreted with caution due to the lack of a control condition. The 2018 U.S. Physical Activity Guidelines recommend that acute exercise could serve as an effective means in facilitating brain health (U.S. Department of Health Human Services, 2008; Chang et al., 2019). As such, results from this study provide further support for the relevance of acute exercise in children with ADHD and provide preliminary evidence for addressing the optimal level of acute exercise intensity to aid inhibitory control.

## Data Availability Statement

The raw data supporting the conclusions of this article will be made available by the authors, without undue reservation.

## Ethics Statement

The studies involving human participants were reviewed and approved by Institutional Review Board at National Taiwan University. Written informed consent to participate in this study was provided by the participants' legal guardian/next of kin.

## Author Contributions

YJT conducted the experiment, analyzed data, and wrote the manuscript. SSH interpreted data and revised the manuscript. CJH and TMH revised critically for important intellectual content. All authors contributed to the article and approved the submitted version.

## Conflict of Interest

The authors declare that the research was conducted in the absence of any commercial or financial relationships that could be construed as a potential conflict of interest.
